# Development of 15 nuclear microsatellite markers in *Deuterocohnia* (Pitcairnioideae; Bromeliaceae) using 454 pyrosequencing

**DOI:** 10.1002/aps3.1147

**Published:** 2018-05-08

**Authors:** Fides Lea Zenk, Cynthia Firmer, Tina Wöhrmann, Luciana Vicente da Silva, Kurt Weising, Bruno Huettel, Gecele Matos Paggi

**Affiliations:** ^1^ Max Planck Institute of Immunobiology and Epigenetics Stübeweg 51 79108 Freiburg im Breisgau Germany; ^2^ Systematics and Morphology of Plants Institute of Biology University of Kassel Heinrich‐Plett‐Str. 40 34132 Kassel Germany; ^3^ Ecology and Conservation Postgraduate Program Federal University of Mato Grosso do Sul State 79070‐900 Campo Grande Brazil; ^4^ Max Planck‐Genome‐centre Cologne Max Planck Institute for Plant Breeding Research Carl‐von‐Linné‐Weg 10 50829 Cologne Germany; ^5^ Biological Sciences Pantanal Campus/Federal University of Mato Grosso do Sul State C.P. 252 79304‐902 Corumbá Brazil

**Keywords:** 454 pyrosequencing, Bromeliaceae, *Deuterocohnia*, genetic differentiation, genetic diversity, microsatellites

## Abstract

**Premise of the Study:**

Microsatellite markers were developed in *Deuterocohnia longipetala* (Bromeliaceae) to investigate species and subspecies boundaries within the genus and the genetic diversity of natural populations.

**Methods and Results:**

We used 454 pyrosequencing to isolate 835 microsatellite loci in *D. longipetala*. Of 64 loci selected for primer design, 15 were polymorphic among 23 individuals of *D. longipetala* and 76 individuals of the heterologous subspecies *D. meziana* subsp. *meziana* and *D. meziana* subsp. *carmineo‐viridiflora*. Twelve and 13 of these loci were also polymorphic in one population each of *D. brevispicata* and *D. seramisiana*, respectively. Numbers of alleles per locus varied from two to 14 in *D. longipetala*, two to 12 in *D. meziana*, one to nine in *D. brevispicata*, and one to 10 in *D. seramisiana*. STRUCTURE analyses clearly separated the taxa from each other.

**Conclusions:**

The 15 new microsatellite markers are promising tools for studying population genetics in *Deuterocohnia* species.

The genus *Deuterocohnia* Mez (Bromeliaceae) includes 17 species that are mainly distributed in the Andes of central South America (Schütz, 2013). It comprises terrestrial or saxicolous plants with thorny leaves in dense rosettes, giving rise to woody, perennial inflorescence axes that are able to bloom for several years (Smith and Downs, 1974; Benzing, 2000). All species are adapted to extremely arid environments such as steep and rocky slopes of the Andes and inter‐Andean valleys, but some also grow on rocky outcrops in lowlands of eastern Bolivia and western Brazil (Schütz, 2013, 2014). Species delimitation within *Deuterocohnia* is often difficult due to hybridization among closely related species and subspecies (Schütz, 2014). Considering data of floral display, seed and floral morphology, and pollinators (Benzing, 2000), it seems that species from *Deuterocohnia* may present a variety of characteristics related to outcrossing. So far, this reproductive system was previously reported for *D. meziana* Kuntze ex Mez, which is self‐incompatible and clonal (Arruda, 2016), and has winged seeds adapted for long‐distance dispersal (Schütz, 2014).

To date, very little is known about the genetic diversity and population structure in any *Deuterocohnia* species. However, this information is important for endangered species like *D. meziana* (Ministério do Meio Ambiente, 2014; Schütz, 2014). It can contribute to our understanding of microevolutionary processes of natural populations, assist in the delimitation of species and subspecies (Palma‐Silva et al., 2011), and help to detect hybridization (Zanella et al., 2016) and to design management and conservation strategies (Ribeiro et al., 2013). Here, we present 15 polymorphic microsatellite loci developed for the genus *Deuterocohnia* using 454 pyrosequencing technology.

## METHODS AND RESULTS

Total DNA was extracted from fresh leaves following the protocol of Tel‐Zur et al. (1999). The source DNA for 454 sequencing was derived from one individual plant of *D. longipetala* (Baker) Mez that was collected along the road from Bermejo to Limal (Bolivia) and that is now cultivated in the greenhouse of the University of Kassel (accession NiSch_06‐068; Appendix 1). We chose this species for microsatellite isolation and primer design because it has the widest distribution range of any *Deuterocohnia* species (Schütz, 2013). Library preparation and pyrosequencing of a 5‐μg DNA aliquot were performed as described by Wöhrmann et al. (2012). Using default settings, 25,827 raw reads with an average length of 337 bp were obtained and imported into the pipeline iQDD (version 1.3; Meglécz et al., 2010); these sequences were also submitted to the National Center for Biotechnology Information's Sequence Read Archive (accession no. SRP126618). From those sequences, we identified 835 perfect repeats with a minimum of seven units for di‐, six for tri‐, five for tetra‐, and four for penta‐ and hexanucleotide repeats, respectively. Sixty‐four microsatellite loci with sufficient flanking sequence and high repeat numbers were selected for PCR primer construction (Appendix 2), following previously described criteria (Wöhrmann et al., 2012).

All primer pairs were initially tested for successful amplification in two individuals each of *D. meziana* subsp. *carmineo‐viridiflora* Rauh (NiSch_06‐007J, NiSch_06‐007M) and *D. brevispicata* Rauh & L. Hrom. (NiSch_06‐040F, NiSch_06‐040M), as well as in one individual each of *D. seramisiana* R. Vásquez, Ibisch & E. Gross (NiSch_06‐045K) and *D. longipetala* (NiSch_06‐068 as a positive control). PCRs were conducted in 12.5‐μL volumes in a T‐Gradient thermocycler (Biometra, Göttingen, Germany) following a touchdown protocol (Wöhrmann et al., 2012). As evidenced by electrophoresis on 1.5% agarose gels, 52 of the 64 primer pairs generated single, distinct PCR products within the expected size range in the positive control (Appendix 2). Forty‐seven primer pairs also performed well in one or more accessions from other *Deuterocohnia* species, and only 12 loci failed in all samples (Appendix 2). Of 22 primer pairs that amplified in all individuals of the test set, 15 were validated by genotyping the full set of 129 samples listed in Appendix 1 (for locus characteristics see Table 1). Fluorescence‐labeled primers were used for PCR, and amplicons were electrophoresed on denaturing 6% polyacrylamide gels in 1× TBE buffer, using an automated sequencer (Li‐Cor 4300 IR^2^; Li‐Cor Biosciences, Lincoln, Nebraska, USA). Fragment sizes were scored with the help of an external size standard as described by Wöhrmann et al. (2012).

**Table 1:** Characteristics of 15 polymorphic microsatellite loci and flanking primer pairs developed for *Deuterocohnia*. Expected allele sizes were inferred from the unique, microsatellite‐containing 454 sequences of *D. longipetala* (accession NiSch_06‐068)

Locus	Primer sequences (5′–3′)	*T*_a_ (°C)	Repeat motif	Expected product size (bp)	GenBank accession no.
ngDeu_5	F: ACTACTTCCAAGACCAAAAGG	55	(GGA)_9_	151	MF838869
	R: TCACTCACTAGAGGGGTACAA				
ngDeu_9	F: GGAACTCGAAGTCGGTGGT	60	(TCG)_10_	189	MF838873
	R: CAATGGCCCAAGAAGAGAAA				
ngDeu_11	F: CGTACGATCGAAAAGCCAAA	61	(GAA)_12_	189	MF838875
	R: ATCAAGTGCGCCTCAAGC				
ngDeu_15	F: GCAAACACAGATGTCGTAAAC	56	(ATCT)_7_	157	MF838879
	R: CTTGGCCTTGCTTATTATTTT				
ngDeu_17	F: CCTTAATGACCTACAGTTTCG	55	(AGAAG)_4_	147	MF838881
	R: CTTGGTTCAGAGGAGGTCTAT				
ngDeu_19	F: GGAGGAGAAGTTGGAGGA	55	(GATCGA)_5_	131	MF838883
	R: CCCTCTTCTCCTTTCCAG				
ngDeu_26	F: AAACCAGAATTACCTCGCGC	59	(TCT)_8_	158	MF838890
	R: CGTGAGTATGTCGGTGGGAT				
ngDeu_43	F: AGATACAAACAAGGAGCAACATG	59	(GA)_12_	150	MF838907
	R: ACGTGCCCTGCTTCTCCAT				
ngDeu_46	F: GCGGGTTAGGGTTAGGGTTA	59	(GA)_12_	200	MF838910
	R: TCTCCCTCTCTTCGTCTCCA				
ngDeu_48	F: ACGACTCCAGTTCTTGCTC	55	(TCT)_6_	165	MF838912
	R: AGAAGTCGTCGGAGAAGTC				
ngDeu_49	F: TGGCGAACATGGACCTCTAG	59	(TCC)_6_	206	MF838913
	R: CGAGTGTTACAGAGCGCTTC				
ngDeu_50	F: TAGACTGAGGCAGGATACAGA	55	(AGT)_6_	144	MF838914
	R: CAGGAAACTGCAAGAAAAGTA				
ngDeu_58	F: GGAGGTTGGAGACGAAGAT	56	(CGC)_7_	149	MF838922
	R: AACCCTAGACACTACGTTGCT				
ngDeu_61	F: ATTCTCACACCCTCCACACA	59	(AAAT)_5_	194	MF838925
	R: AAAGAACAAGCTGGACCACG				
ngDeu_63	F: TAGGCTGTCGGTTTGGATGT	59	(TCTCT)_4_	197	MF838927
	R: AGAAACTCTCTCCCTTGTTCTCT				

Note

*T*_a_ = optimal annealing temperature (averaged over both values).

Population genetic parameters are compiled in Table 2. Allele numbers as well as observed (*H*_o_) and expected (*H*_e_) heterozygosity values were determined with ARLEQUIN version 3.11 (Excoffier et al., 2005). Wright's inbreeding coefficients (*F*_IS_) and deviations from Hardy–Weinberg equilibrium (HWE) were calculated with GENEPOP (Raymond and Rousset, 1995). All 15 loci proved to be polymorphic in *D. longipetala* and in *D. meziana*, whereas three and two loci, respectively, were monomorphic in *D. brevispicata* and *D. seramisiana*. Altogether 80 alleles were detected in 23 individuals of *D. longipetala* from various localities, showing mean heterozygosity values of 0.44 (*H*_o_) and 0.66 (*H*_e_). A total of 68 alleles were detected in 76 individuals of *D. meziana*, represented by *D. meziana* subsp. *carmineo‐viridiflora* (two populations, *n* = 28) and *D. meziana* subsp. *meziana* (five populations, *n* = 48), and the overall number of alleles ranged from two to 12. In the *D. brevispicata* population (*n* = 13), mean heterozygosity values of 0.39 (*H*_o_) and 0.50 (*H*_e_) over all loci were obtained. Finally, one to 10 alleles per locus were found in the 17 samples of the *D. seramisiana* population. Mean heterozygosity values in this species were 0.47 (*H*_o_) and 0.51 (*H*_e_). Mean *F*_IS_ values ranged from a minimum of 0.11 for *D. meziana* subsp. *carmineo‐viridiflora* to a maximum of 0.32 for *D. longipetala* (Table 2, Appendix 1). Significant deviations from HWE were observed at 11 loci in *D. longipetala*, at three loci each in *D. meziana* subsp. *carmineo‐viridiflora* and *D. brevispicata*, at four loci in *D. meziana* subsp. *meziana*, and at two loci in *D. seramisiana* (Table 2).

**Table 2:** Population genetic parameters determined in *Deuterocohnia longipetala*,* D. meziana* subsp. *carmineo‐viridiflora*,* D. meziana* subsp. *meziana*,* D. brevispicata*, and *D. seramisiana* across 15 polymorphic microsatellite markers.^a^

	*D. longipetala* (*n* = 23)				*D. meziana* subsp. *carmineo‐viridiflora* (*n* = 28)				*D. meziana* subsp. *meziana* (*n* = 48)					*D. brevispicata* (*n* = 13)				*D. seramisiana* (*n* = 17)				All samples (*n* = 129)		
Locus	*A*	*H*_o_	*H*_e_	*F*_IScv_ b	*A*	*H*_o_	*H*_e_	*F*_ISmm_ b	*A*	*H*_o_	*H*_e_	*F*_IS_ b	*A*_mez_	*A*	*H*_o_	*H*_e_	*F*_IS_ b	*A*	*H*_o_	*H*_e_	*F*_IS_ b	*A*	*H*_o_	*H*_e_
ngDeu_5	5	0.57	0.79	0.29*	6	0.71	0.62	−0.16^ns^	8	0.71	0.83	0.15^ns^	8	6	0.62	0.69	0.11^ns^	6	0.71	0.63	−0.12^ns^	11	0.67	0.86
ngDeu_9	8	0.52	0.84	0.39**	6	0.50	0.47	−0.07^ns^	5	0.49	0.65	0.25*	6	5	0.85	0.77	−0.11^ns^	6	0.75	0.82	0.08^ns^	13	0.57	0.80
ngDeu_11	14	0.83	0.92	0.11***	5	0.79	0.78	−0.01^ns^	1	—	—	—	5	9	0.85	0.90	0.06*	10	0.94	0.86	−0.10^ns^	19	0.53	0.73
ngDeu_15	8	0.30	0.74	0.59***	3	0.50	0.52	0.04^ns^	2	0.11	0.18	0.40*	3	6	0.54	0.75	0.29**	1	—	—	—	10	0.26	0.75
ngDeu_17	3	0.26	0.34	0.23^ns^	2	0.30	0.39	0.25^ns^	1	—	—	—	2	2	0.08	0.08	—	1	—	—	—	3	0.12	0.36
ngDeu_19	9	0.39	0.86	0.55***	5	0.61	0.63	0.04^ns^	3	0.26	0.38	0.33^ns^	6	8	0.38	0.87	0.57***	5	0.53	0.58	0.09^ns^	13	0.41	0.83
ngDeu_26	2	0.70	0.49	−0.44^ns^	2	0.20	0.18	−0.09^ns^	3	0.05	0.05	−0.01^ns^	3	1	—	—	—	4	0.76	0.57	−0.37^ns^	5	0.33	0.34
ngDeu_43	4	0.48	0.70	0.32**	3	0.70	0.66	−0.06^ns^	1	0.07	0.07	−0.02^ns^	4	4	0.86	0.74	−0.18^ns^	3	0.18	0.27	0.34^ns^	9	0.35	0.75
ngDeu_46	8	0.65	0.85	0.24***	10	0.76	0.85	0.11^ns^	8	0.42	0.82	0.49***	12	4	0.67	0.71	0.06^ns^	7	0.76	0.81	0.05^ns^	15	0.61	0.87
ngDeu_48	3	0.43	0.50	0.14^ns^	3	0.21	0.38	0.46*	2	0.14	0.17	0.18^ns^	3	3	0.43	0.56	0.25^ns^	4	0.24	0.53	0.56**	4	0.25	0.48
ngDeu_49	3	0.39	0.58	0.33**	2	0.52	0.51	−0.02^ns^	1	—	—	—	2	2	0.23	0.47	0.52^ns^	3	0.41	0.47	0.13^ns^	3	0.28	0.52
ngDeu_50	3	0.39	0.50	0.22^ns^	3	0.68	0.55	−0.24^ns^	2	0.00	0.50	1.00***	3	2	0.00	0.21	1.00^ns^	2	0.29	0.52	0.44^ns^	3	0.28	0.64
ngDeu_58	4	0.30	0.72	0.58***	4	0.21	0.58	0.65***	2	0.14	0.13	−0.05^ns^	4	1	—	—	—	2	0.36	0.30	−0.18^ns^	7	0.22	0.68
ngDeu_61	4	0.13	0.61	0.79***	5	0.20	0.55	0.64***	2	0.08	0.07	−0.03^ns^	5	1	—	—	—	5	0.43	0.64	0.34*	7	0.16	0.66
ngDeu_63	2	0.26	0.50	0.49*	2	0.21	0.19	−0.10^ns^	1	—	—	—	2	4	0.42	0.71	0.42^ns^	3	0.76	0.63	−0.22^ns^	5	0.24	0.44
Mean	5.3	0.44	0.66	0.32	4.1	0.47	0.52	0.11	3.6	0.22	0.35	0.24	4.5	3.9	0.39	0.50	0.28	4.1	0.47	0.51	0.12	8.5	0.35	0.65
Total	80				61				42				68	58				62				127		

Note

*A* = number of alleles; *A*_mez_ = number of alleles across all *D. meziana* samples; *F*_IS_ = inbreeding coefficient; *F*_IScv_ = inbreeding coefficient observed in *D. meziana* subsp. *carmineo‐viridiflora*;* F*_ISmm_ = inbreeding coefficient observed in *D. meziana* subsp. *meziana*;* H*_e_ = expected heterozygosity; *H*_o_ = observed heterozygosity; *n* = number of individuals tested.

^a^ Locality and voucher information are provided in Appendix 1.

^b^ *P* value of *F*_IS_ (***< 0.001, **< 0.01, *< 0.05, ns = not significant).

**Figure 1:**
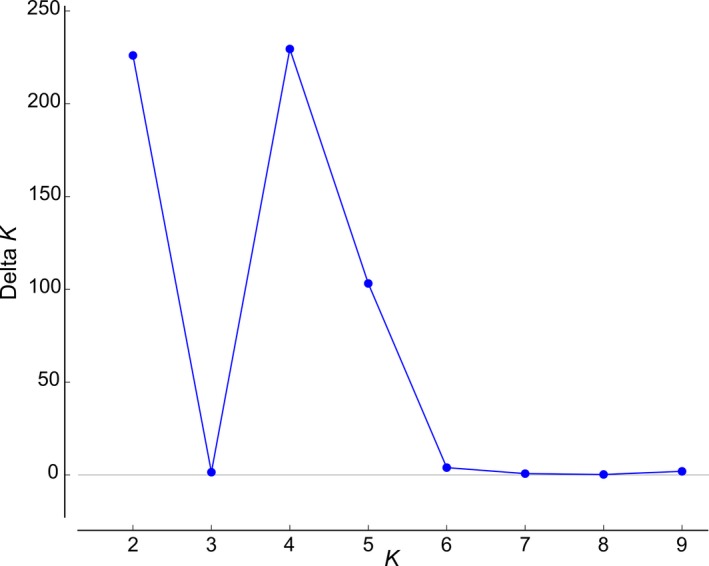
STRUCTURE results for natural populations of *Deuterocohnia meziana* subsp. *carmineo‐viridiflora*,* D. meziana* subsp. *meziana*,* D. brevispicata*,* D. seramisiana*, and *D. longipetala* from central Bolivia and western Brazil showing the *K* graph from STRUCTURE HARVESTER indicating a maximum at *K* = 4. Delta *K* = mean(|L’’(K)|) / sd(L(K)).

**Figure 2:**
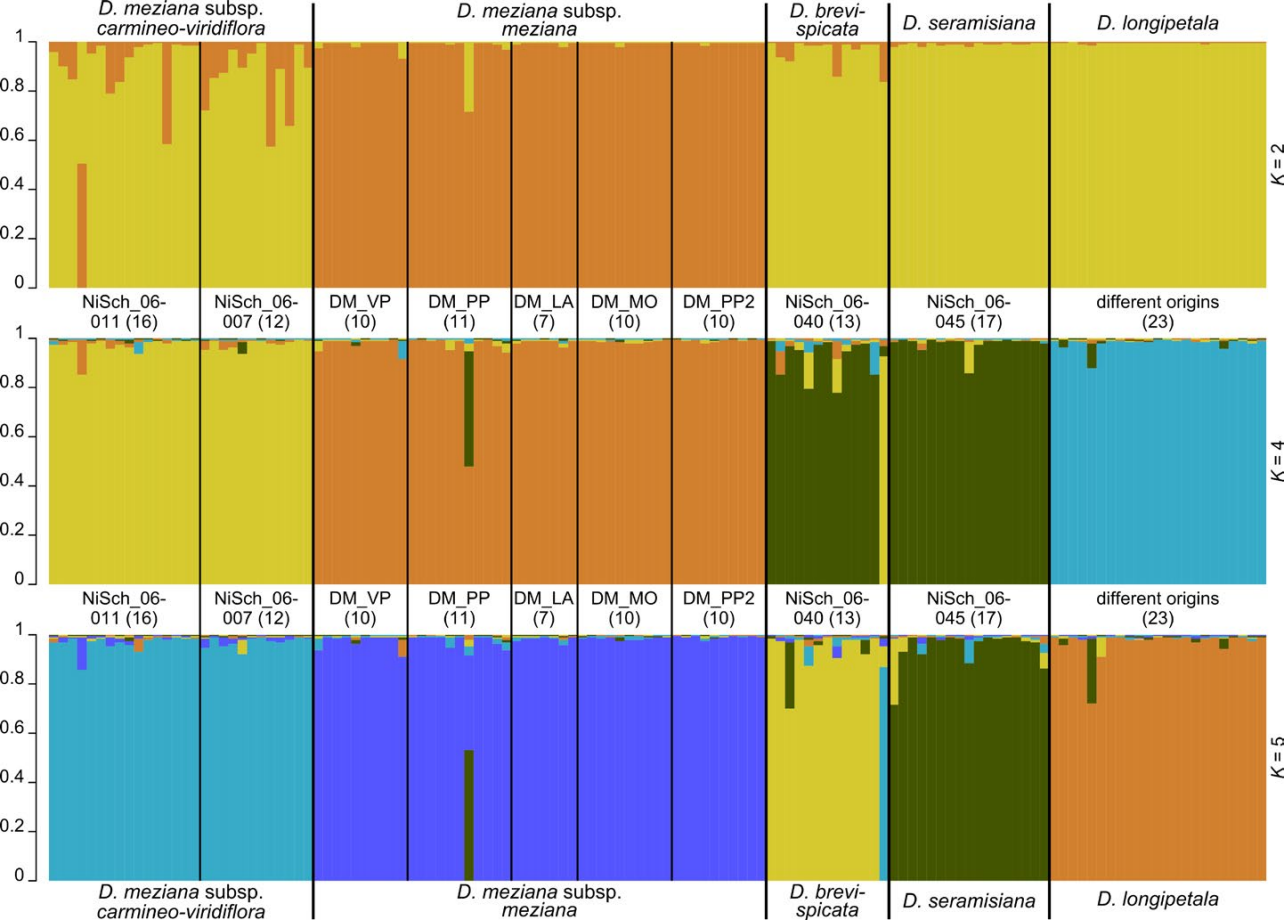
STRUCTURE results for natural populations of *Deuterocohnia meziana* subsp. *carmineo‐viridiflora*,* D. meziana* subsp. *meziana*,* D. brevispicata*,* D. seramisiana*, and *D. longipetala* from central Bolivia and western Brazil showing the bar plot with individual assignments to groups for *K* = 2 (upper panel), *K* = 4 (middle panel), and *K* = 5 (lower panel). Populations and numbers of samples per population are depicted between bar plots.

To evaluate the potential of microsatellite markers for distinguishing between closely related taxa, a Bayesian cluster analysis was performed on a set of 129 plants comprising all samples from the two subspecies of *D. meziana*,* D. brevispicata*,* D. seramisiana*, and *D. longipetala*, using the program STRUCTURE version 2.3.4 (Pritchard et al., 2000). For the determination of the most appropriate number of genetic clusters (*K* value), the analysis was run for 1,000,000 generations in the burn‐in period and for 100,000 generations in the Markov chain Monte Carlo simulation analyses after burn‐in. Ten repetitions for each *K* (1 ≤ *K* ≤ 10) were performed, and the admixture level for each individual (Q) was also inferred. By calculating the Δ*K* statistic using STRUCTURE HARVESTER version 0.6.94 (Earl and von Holdt, 2012), the most likely number of clusters was identified to be four, closely followed by two and five (Fig. 1). Final plots were visualized in STRUCTURE PLOT version 2.0 (Ramasamy et al., 2014). For the three estimates of *K* (2, 4, and 5), there is a clear division among one cluster composed of all *D. meziana* subsp. *meziana* samples (Fig. 2). For *K* = 4, there is a second cluster containing all *D. meziana* subsp. *carmineo‐viridiflora* plants, a third cluster combining all samples from *D. brevispicata* and *D. seramisiana*, and a fourth containing all samples from *D. longipetala* (Fig. 2, middle panel). Assuming *K* = 5, *D. brevispicata* and *D. seramisiana* also become clearly separated from each other (Fig. 2, lower panel).

## CONCLUSIONS

The 15 microsatellite markers developed from 454 sequences of *D. longipetala* revealed moderate levels of genetic diversity in the source species as well as in three heterologous *Deuterocohnia* taxa investigated. Whereas the two subspecies of *D. meziana* were surprisingly well separated from each other, the distinction between *D. brevispicata* and *D. seramisiana* was less pronounced, suggesting some ongoing gene flow among populations of these two species. The microsatellite markers developed here are promising tools for the study of population genetics, phylogeography, and the cohesion and delimitation of species and subspecies in *Deuterocohnia*. Genetic data generated by these markers will also provide important guidelines for designing management and conservation strategies in endangered species like *D. meziana*.

## APPENDIX 1 Geographical origins and voucher information of the 129 *Deuterocohnia* individuals analyzed in the present study.

**Table:**

Taxon	Locality/source	Plant ID/voucher	Herbarium^a^	*n*	Geographic coordinates
*D. meziana* Kuntze ex Mez subsp*. meziana*	Brazil, Mato Grosso do Sul	322	COR	10	19.95225°S, 57.332841°W
*D. meziana* subsp. *meziana*	Brazil, Mato Grosso do Sul	LA 1	COR	11	19.143316°S, 57.381266°W
*D. meziana* subsp. *meziana*	Brazil, Mato Grosso do Sul	327	COR	7	19.141138°S, 57.384622°W
*D. meziana* subsp. *meziana*	Brazil, Mato Grosso do Sul	SO 1	COR	10	19.164783°S, 57.315494°W
*D. meziana* subsp. *meziana*	Brazil, Mato Grosso do Sul	AT 722	COR	10	19.178080°S, 57.377043°W
*D. meziana* subsp. *carmineo‐viridiflora* Rauh	Bolivia, Santa Cruz de La Sierra	NiSch_06‐011	FR	16	18.14867°S, 63.92992°W
*D. meziana* subsp. *carmineo‐viridiflora*	Bolivia, Santa Cruz de La Sierra	NiSch_06‐007	FR	12	18.01537°S, 64.10005°W
*D. brevispicata* Rauh & L. Hrom.	Bolivia, Chuquicasaca	NiSch_06‐040	FR	13	19.66250°S, 64.03533°W
*D. seramisiana* R. Vásquez, Ibisch & E. Gross	Bolivia, Chuquicasaca	NiSch_06‐045	FR	17	19.14432°S, 64.51910°W
*D. longipetala* (Baker) Mez (N 116)	Unknown	285‐01‐89‐83	B	1	Unknown
*D. longipetala* (N 273)	Unknown	WT 5165	WU	1	30.50°S, 66.35°W
*D. longipetala* (N 127)	Argentina	WT sn	B	1	Unknown
*D. longipetala* (N 245)	Argentina	WT sn	HEID	1	Unknown
*D. longipetala* (N 269)	Argentina, Córdoba	WT 5025	WU	1	Unknown
*D. longipetala* (N 270)	Argentina, Córdoba	WT 5038	WU	1	30.50°S, 64.35°W
*D. longipetala* (N 274)	Argentina, Córdoba	WT 5221	WU	1	Unknown
*D. longipetala* (N 260)	Argentina, Jujuy	WT 10082 a	WU	1	23.54°S, 65.28°W
*D. longipetala* (N 264)	Argentina, Jujuy	WT 10126	WU	1	24.29°S, 65.1730°W
*D. longipetala* (N 131)	Argentina, La Rioja	Leuenberger 4478a	HEID	1	30.4707°S, 66.9048°W
*D. longipetala* (N 271)	Argentina, La Rioja	WT 5089	WU	1	29.00°S, 67.28°W
*D. longipetala* (N 280)	Argentina, La Rioja	sn	WU	1	29.10°S, 67.30°W
*D. longipetala* (N 284)	Argentina, La Rioja	WT 5068	WU	1	29.54°S, 67.09°W
*D. longipetala* (N 272)	Argentina, San Juan	WT 5131	WU	1	30.3830°S, 67.29°W
*D. longipetala* (N 208)	Argentina, Salta	NiSch_06‐118	LIL	1	25.4046°S, 65.4127°W
*D. longipetala* (N 210)	Argentina, Salta	NiSch_06‐124	LIL	1	Unknown
*D. longipetala* (N 257)	Argentina, Tucumán	WT 10045	WU	1	26.16°S, 65.30°W
*D. longipetala* (N 259)	Argentina, Tucumán	WT 10050	WU	1	26.18°S, 65.35°W
*D. longipetala* (N 267)	Argentina, Tucumán	WT 10249	WU	1	Unknown
*D. longipetala* (N 175)	Bolivia, Tarija	NiSch_06‐066	FR	1	22.2839°S, 64.2859°W
*D. longipetala* (N 176)	Bolivia, Tarija	NiSch_06‐067	FR	1	22.2929°S, 64.2754°W
*D. longipetala* (N 276)	Bolivia, Tarija	WT 79	WU	1	21.25°S, 63.58°W
*D. longipetala* (positive control)	Bolivia, Tarija	NiSch_06‐068	KAS	1	22.57043°S, 64.41242°W

Notes

AT = Adriana Takahasi; LA = Lescano Almeida; NiSch = Nicole Schütz; *n* = number of samples; SO = Silvia Ortiz; WT = Walter Till.

^a^ Herbaria acronyms are per Index Herbariorum (http://sweetgum.nybg.org/science/ih/).

## APPENDIX 2 Characteristics of primer pairs designed on the basis of *Deuterocohnia longipetala* microsatellite loci (accession NiSch_06‐068).

**Table:**

Locus	GenBank accession no.	Repeat motif	Expected product size (bp)	Forward primer (5′–3′)	Reverse primer (5′–3′)	*T*_a_ (°C)	Efficiency of PCR amplification^a^						
							1	2	3	4	5	6	#
ngDeu_1	MF838865	(TA)_12_	103	GGATGTTGATGCAAGGTG	CGATTAAGCTAACAAAATACAAC	54	(+)	(+)	(+)	—	—	+	4
ngDeu_2	MF838866	(AC)_14_	166	ATTAAGTCTAGCGAAGCTGGT	AAGTTGGCGTCATATTTTAAC	55	—	—	—	—	—	—	—
ngDeu_3	MF838867	(TG)_15_	118	AAATCGCTTGTTAAACCCTAT	GCTAGAGTTACTAAGAGCAACCA	55	—	—	—	—	(+)	+	2
ngDeu_4	MF838868	(GA)_15_	141	ACCTGGTATTTGAGTGGTTCT	CTTCTTCCTCCTCACTACTCC	55	—	—	—	—	—	—	—
ngDeu_5*	MF838869	(GGA)_9_	151	ACTACTTCCAAGACCAAAAGG	TCACTCACTAGAGGGGTACAA	55	+	+	+	+	+	+	6
ngDeu_6	MF838870	(GCC)_9_	129	CCTTCGCTTCTACCTCTTTC	CCTTGCACCGCCATAGAT	58	—	—	—	—	—	—	—
ngDeu_7	MF838871	(TTC)_10_	149	CACGAATACGCCGACTAC	CGACGGTAAAAACAGTAAACA	55	—	—	(+)	(+)	(+)	(+)	4
ngDeu_8	MF838872	(AAT)_10_	151	ATTAAAACAACCACGTCAAAG	GGGCTACAACAGATGTAACAG	55	—	—	—	—	—	—	—
ngDeu_9*	MF838873	(TCG)_10_	189	GGAACTCGAAGTCGGTGGT	CAATGGCCCAAGAAGAGAAA	60	+	+	+	+	+	+	6
ngDeu_10	MF838874	(GGA)_10_	146	GCGACGTTGTAATTCACTATC	CGCATACATCACCTCTTCTT	55	—	—	—	—	—	—	—
ngDeu_11*	MF838875	(GAA)_12_	189	CGTACGATCGAAAAGCCAAA	ATCAAGTGCGCCTCAAGC	61	+	+	+	+	+	+	6
ngDeu_12	MF838876	(AAT)_17_	152	GTGCAGATCTTACACCTCCTA	CTCCAACCAAGAGTTATTATTTC	54	+	+	+	(+)	+	+	6
ngDeu_13	MF838877	(TAA)_9_	165	AAGATTTAGACCCCACTCCTT	CATAGAGTTCCATTTCGTTTG	56	—	—	+	—	—	+	2
ngDeu_14	MF838878	(ATAC)_6_	134	TAGATGATTTTTGCAGGGATA	ACGAGAACATTTATGCGAGTA	55	—	—	—	—	—	+	1
ngDeu_15*	MF838879	(ATCT)_7_	157	GCAAACACAGATGTCGTAAAC	CTTGGCCTTGCTTATTATTTT	56	+	+	+	+	+	+	6
ngDeu_16	MF838880	(TTAT)_9_	144	GTGCTTTCGATTTGTAGACAG	TTGGTCCCTCTTCTCTCATA	55	—	—	—	—	—	+	1
ngDeu_17*	MF838881	(AGAAG)_4_	147	CCTTAATGACCTACAGTTTCG	CTTGGTTCAGAGGAGGTCTAT	55	+	+	+	+	+	+	6
ngDeu_18	MF838882	(AAAAAT)_5_G(ATA)_17_	170	ACAACCTAGGTCTCGAAGAAG	TACAAGCTGTTGTTGAAGGAT	55	(+)	+	(+)	—	+	+	5
ngDeu_19*	MF838883	(GATCGA)_5_	131	GGAGGAGAAGTTGGAGGA	CCCTCTTCTCCTTTCCAG	55	+	+	+	+	+	+	6
ngDeu_20	MF838884	(CCTCGC)_5_	192	CATCTTCCTTCTTCCTTCCT	TATAAGGAGAAGATCGTGGTG	55	+	+	+	(+)	+	(+)	6
ngDeu_21	MF838885	(GA)_12_	136	CTCAAGATAGAGTGTTAGTCCAA	TTATATAAAACCTAGCCGTCA	53	—	—	—	—	—	+	1
ngDeu_22	MF838886	(AT)_13_	159	CTGTAAGAGTTGGAACTACGC	TGATGGTGAGATTACCTCAAT	55	(+)	+	+	+	(+)	+	6
ngDeu_23	MF838887	(CT)_15_	145	AACCCTAAAAGACTTCTCTCG	GGTAGGTACGGTAGGTAGGAG	55	—	—	(+)	(+)	+	+	4
ngDeu_24	MF838888	(TCC)_8_	102	ATCCTCGTCGTCCTCATC	GTAGATCGCTACCGCAAGAT	56	—	—	—	—	—	—	—
ngDeu_25	MF838889	(CGC)_8_	164	CTCTTCTTCTTCCCCTTCC	CTGGAGAATTGCCAGATG	55	—	+	+	+	+	+	5
ngDeu_26*	MF838890	(TCT)_8_	158	AAACCAGAATTACCTCGCGC	CGTGAGTATGTCGGTGGGAT	59	+	+	+	+	+	+	6
ngDeu_27	MF838891	(ATT)_8_	167	CGATTGTGAACAGCTTATGAT	CACTTTCTTCTTGATTTTTGG	55	+	+	+	+	+	+	6
ngDeu_28	MF838892	(ATT)_9_	150	TCGAAAATCTAACAGCATCAT	AACCGCACATCTCTTAATCTA	55	—	—	+	+	—	+	3
ngDeu_29	MF838893	(CTT)_9_	164	ATTTTTGTCCCTCTTCATGTT	GACGACAAAGAAGAATCAGAA	55	(+)	—	(+)	—	+	+	4
ngDeu_30	MF838894	(TTC)_9_	132	ATTTAATTGCTTGGAGGAAGT	GAAGCTTAAAGGGGAATATCA	55	(+)	(+)	+	+	+	+	6
ngDeu_31	MF838895	(CTT)_10_	146	GCGAAGCGGAAGGTTAAG	TAGTGAACCTGTGAAATGACC	57	—	—	—	—	—	—	—
ngDeu_32	MF838896	(TAA)_12_	187	CAAAAGAGAAATAGCGTGTCA	CACATGGTGGTATTTGAAGTT	56	—	—	—	(+)	—	(+)	2
ngDeu_33	MF838897	(ATA)_8_	156	TTCACAAGGGAAATAGTGAAA	CACTCACCATCATCGACTATC	55	—	—	+	—	+	+	3
ngDeu_34	MF838898	(TATT)_6_	135	GGCAAAAATGAAAATGTGAT	ATACAGCAAAACAACATTCCA	56	+	—	+	—	+	+	4
ngDeu_35	MF838899	(TTCT)_6_	134	AGATGATCTTCCTCTTCTTGG	GACAGGAGAGAGAAAGGAAAG	55	—	(+)	—	—	(+)	+	3
ngDeu_36	MF838900	(AGGC)_6_	143	AGTCGACGATCTAATCCATCT	GAGGGGGAAAAGAAAAGTTAT	56	—	—	+	—	+	+	3
ngDeu_37	MF838901	(GGTTC)_4_	148	GGAATGCCATACTTATTCACA	ATGACGCCGAACCCGAAC	60	—	—	—	—	—	+	1
ngDeu_38	MF838902	(AAATC)_4_	147	GAATAGATAGGGGTTGAGGAA	TGATTACAAATCTGGAGGAAG	55	(+)	(+)	+	+	+	+	6
ngDeu_39	MF838903	(GTTAGG)_5_	150	GAGGAGGAGGAAGGGAAG	GACCATCCGAGAAGATGAG	56	—	(+)	(+)	(+)	(+)	+	5
ngDeu_40	MF838904	(CGCCTC)_5_	154	GTGTGCTCACTCTCTTCTGTC	CTTTTGTTTGAACGAACGAC	56	—	—	—	—	—	+	1
ngDeu_41	MF838905	(AG)_7_	156	TAAACAAACTCCTCTCAACCA	GAGGGTGGAGACTTAGAGAAG	55	+	+	+	+	(+)	(+)	6
ngDeu_42	MF838906	(AT)_7_	203	ACTCTCTGGGAAGTAGAGCAT	AAAAGGAGGGTGAGGTTACTA	55	(+)	(+)	(+)	(+)	—	+	5
ngDeu_43*	MF838907	(GA)_12_	150	AGATACAAACAAGGAGCAACATG	ACGTGCCCTGCTTCTCCAT	59	+	+	+	+	+	+	6
ngDeu_44	MF838908	(CG)_7_	196	TTGAAAAGACTCGGCGTTCA	TGCCGTTAGTGTTGTGTGTG	59	—	—	—	—	—	—	—
ngDeu_45	MF838909	(AT)_9_	158	CACTATTTGTCACGCCCCAG	CGTGCAAATACAGGGGAGAC	59	+	+	+	+	—	+	5
ngDeu_46*	MF838910	(GA)_12_	200	GCGGGTTAGGGTTAGGGTTA	TCTCCCTCTCTTCGTCTCCA	59	+	+	+	+	+	+	6
ngDeu_47	MF838911	(CA)_13_	155	TCTTCTTTCGAGATATGTTGCCT	GTTTAGGAAGGTAGCTCGCG	58	—	—	—	—	—	—	—
ngDeu_48*	MF838912	(TCT)_6_	165	ACGACTCCAGTTCTTGCTC	AGAAGTCGTCGGAGAAGTC	55	+	+	+	+	+	+	6
ngDeu_49*	MF838913	(TCC)_6_	206	TGGCGAACATGGACCTCTAG	CGAGTGTTACAGAGCGCTTC	59	+	+	+	+	+	+	6
ngDeu_50*	MF838914	(AGT)_6_	144	TAGACTGAGGCAGGATACAGA	CAGGAAACTGCAAGAAAAGTA	55	+	+	+	+	+	+	6
ngDeu_51	MF838915	(AGT)_6_	195	AGGGAGAGCATTATGTGGCA	GCACACACTAGCAGACAGGA	59	+	+	+	+	—	+	5
ngDeu_52	MF838916	(CCG)_6_	133	AGTCGGTATTGGGACGAG	GACCGTAGTCGTAGGTCGT	56	(+)	(+)	(+)	—	—	+	4
ngDeu_53	MF838917	(CCG)_6_	171	ATCACCAGATGAGGTAGGAAG	CTGGGAGCGATAGGGTTC	57	—	—	—	—	—	—	—
ngDeu_54	MF838918	(CGC)_6_	158	AGAGGAAGAAGATGACGATTC	AGGAGCGTAGGTTCAACAC	55	—	+	—	—	—	+	2
ngDeu_55	MF838919	(CGG)_6_	271	TCCCTGTGGTTTGGATCTGT	TGTTGTTGTAGTCGATGATCCG	59	(+)	(+)	—	+	(+)	+	4
ngDeu_56	MF838920	(GCG)_6_	142	GATGTAGAACGCCCCTCT	ACTTCGGAGGAAACAATAGTC	55	—	—	—	—	—	—	—
ngDeu_57	MF838921	(TTC)_7_	182	CAAGGATGGCATCGTCGC	ACGGTGAACCTGTGAAATGAC	59	—	—	—	(+)	(+)	+	3
ngDeu_58*	MF838922	(CGC)_7_	149	GGAGGTTGGAGACGAAGAT	AACCCTAGACACTACGTTGCT	56	+	+	+	+	+	+	6
ngDeu_59	MF838923	(AAT)_15_	154	GAAAATTTATTGAGCATCGTG	TTGGATGTTGCTAATATTCCT	55	—	—	—	—	—	—	—
ngDeu_60	MF838924	(ATA)_21_	232	GCTACAGACGTGAGACAACC	CATGATCTTCAGGTCCGCC	58	+	+	—	+	+	+	5
ngDeu_61*	MF838925	(AAAT)_5_	194	ATTCTCACACCCTCCACACA	AAAGAACAAGCTGGACCACG	59	+	+	+	+	+	+	6
ngDeu_62	MF838926	(CCTC)_5_	151	AGCTCCATCCTATAATCAGTCCA	GCCGGATCTAGGGGTTTCC	59	—	—	—	—	+	(+)	2
ngDeu_63*	MF838927	(TCTCT)_4_	197	TAGGCTGTCGGTTTGGATGT	AGAAACTCTCTCCCTTGTTCTCT	59	+	+	+	+	+	+	6
ngDeu_64	MF838928	(CTCCT)_4_	240	ACCCAGTAGTCCATTACCCA	AGATTGAGCTGAGGAACCCA	58	+	+	—	—	+	+	4

Note

+ = one distinct PCR product observed on 1.5% agarose gels; (+) = weak bands; — = no PCR product observed; # = number of accessions for which a successful PCR amplification could be detected.

^a^ Success of PCR amplification in a test set consisting of six *Deuterocohnia* individuals: 1 = NiSch_06‐007J, 2 = NiSch_06‐007M (both individuals of *D. meziana* subsp. *carmineo‐viridiflora*); 3 = NiSch_06‐040F, 4 = NiSch_06‐040M (both individuals of *D. brevispicata*); 5 = NiSch_06‐045K (*D. seramisiana*); 6 = NiSch_06‐068 (*D. longipetala* as positive control).

^*^ Primer pairs used in the present study (see also Tables 1 and 2).
